# Involvement of the Niacin Receptor GPR109a in the Local Control of Glucose Uptake in Small Intestine of Type 2 Diabetic Mice

**DOI:** 10.3390/nu7095352

**Published:** 2015-09-08

**Authors:** Tung Po Wong, Leo Ka Yu Chan, Po Sing Leung

**Affiliations:** School of Biomedical Sciences, Faculty of Medicine, The Chinese University of Hong Kong, Shatin, Hong Kong, China; E-Mails: wongtp@cuhk.edu.hk (T.P.W.); leokychan@link.cuhk.edu.hk (L.K.Y.C.)

**Keywords:** enterocytes, jejunum, glucose transport, SGLT1, GLUT2, Caco-2, hyperglycemia, hyperlipidemia

## Abstract

Niacin is a popular nutritional supplement known to reduce the risk of cardiovascular diseases by enhancing high-density lipoprotein levels. Despite such health benefits, niacin impairs fasting blood glucose. In type 2 diabetes (T2DM), an increase in jejunal glucose transport has been well documented; however, this is intriguingly decreased during niacin deficient state. In this regard, the role of the niacin receptor GPR109a in T2DM jejunal glucose transport remains unknown. Therefore, the effects of diabetes and high-glucose conditions on GPR109a expression were studied using jejunal enterocytes of 10-week-old *m+/db* and *db/db* mice, as well as Caco-2 cells cultured in 5.6 or 25.2 mM glucose concentrations. Expression of the target genes and proteins were quantified using real-time polymerase chain reaction (RT-PCR) and Western blotting. Glucose uptake in Caco-2 cells and everted mouse jejunum was measured using liquid scintillation counting. 10-week T2DM increased mRNA and protein expression levels of GPR109a in jejunum by 195.0% and 75.9%, respectively, as compared with the respective *m+/db* control; high-glucose concentrations increased mRNA and protein expression of GPR109a in Caco-2 cells by 130.2% and 69.0%, respectively, which was also confirmed by immunohistochemistry. In conclusion, the enhanced GPR109a expression in jejunal enterocytes of T2DM mice and high-glucose treated Caco-2 cells suggests that GPR109a is involved in elevating intestinal glucose transport observed in diabetes.

## 1. Introduction

Diabetes mellitus is classically one of the most common chronic metabolic disorders, which is characterized by a plethora of clinical complications; they include, but are not limited to, cardiovascular disease, end-stage renal diseases, diabetic foot disorders, and blindness. One of the most notable clinical manifestations of diabetic patients is dyslipidemia, which is the major risk factor for cardiovascular disease in type 2 diabetes mellitus (T2DM). It has been reported that diabetics have significantly higher coronary-event-associated deaths than the non-diabetics [1]. Furthermore, diabetic individuals without known clinical coronary heart disease (CHD) are exposed to comparable risk of mortality compared to those non-diabetics who have experienced a myocardial infarction [2], thus leading to the recognition of diabetes as a CHD-risk-equivalent condition [3]. 

In order to address this issue, scientists have taken strides to develop novel therapeutic agents that ameliorate cardiovascular disease by specifically targeting dyslipidemia in diabetic patients. In recent years, niacin, also known as nicotinic acid or Vitamin B3, has been a popular nutritional supplement; in fact, it is widely used to treat cardiovascular disease which is associated with dyslipidemia by lowering the level of low-density lipoprotein and triglycerides, while raising the level of high-density lipoprotein simultaneously [4]. Notwithstanding the existence of these health benefits, niacin has been shown to induce glucose intolerance in patients, as evidenced by significant increases in blood glucose levels (5.83 mg/dL) after three years of niacin treatment in euglycemic patients (9.88 mg/dL for niacin *vs.* 4.05 mg/dL for without niacin) [5]. Meanwhile, the incidence rate of impaired fasting blood glucose was increased by 29%. In patients suffering from hyperglycemia, high doses of niacin also increased their level of Hemoglobin A1c by about 0.3% [6]. It is recognized that oral niacin elevated blood glucose levels and thereby deteriorated diabetes in some patients; however, the precise mechanism still remains unknown [7]. In contrast, a lower V_max_ for jejunal glucose uptake *vs.* control group was observed in rats fed with niacin deficient diet [8]. Niacin deficiency also significantly lowers the active electrogenic absorption of glucose across jejunal mucosa [9]. Collectively, these findings have shown that niacin, to a certain extent, might not be beneficial in terms of its adverse effects on glucose homeostasis observed in diabetic patients. 

Of interest in this context is our recent data showing that such a niacin-induced hyperglycemia is probably mediated via the activation of niacin receptor GPR109A-mediated ROS-PPARγ-UCP2 (reactive oxygen species-peroxisome proliferator-activated receptor-γ-uncoupling protein 2) signaling pathway in the pancreatic islet β-cells [10]. In addition, previous studies have shown that niacin exerts its effects by binding to its specific G protein-coupled receptors HM74 (GPR109B) and its homologues HM74A (GPR109A) [11,12,13]. In terms of its potency, niacin activates GPR109A and GPR109B with half maximal effective concentration (EC_50_) values of 0.1 mM and 100 mM, respectively [14]. Both GPR109A and GPR109B have been expressed in the human colon and localized to the apical membrane of colonocytes [15]. In mice, GPR109A is expressed in both small and large intestines [12,16]. Despite these findings, the role of GPR109A in jejunal glucose uptake is still elusive. Interestingly, jejunal glucose transport via both sodium-dependent glucose transporter (SGLT1) and glucose transporter 2 (GLUT2) is greatly increased in early diabetes [17]. In this respect, SGLT1 is a high-affinity, low-capacity transporter localized at the brush border membrane (BBM) of jejunal enterocytes, which is responsible for active uptake of glucose from the intestinal lumen; however, GLUT2 is a glucose transporter normally situated on the basolateral membrane of the jejunal enterocytes but it is translocated and inserted into the BBM during enhanced SGLT1-mediated glucose uptake [18,19,20]. In this way, glucose transport capability of GLUT2 can be up to three times greater than that of SGLT1 [21].

Given the fact that dietary glucose is a critical component in determining our blood glucose homeostasis and in light of the above-mentioned findings, the present study was designed to unravel the undiscovered role of niacin in the regulation of intestinal glucose uptake, with particular emphasis on T2DM, and its potential interaction with the intestinal glucose transporters, SGLT1 and GLUT2, thus its consequence on glucose homeostasis.

## 2. Experimental Section 

### 2.1. Animals 

4- to 12-week-old male *m+/db* and *db/db* mice in this study were used and supplied by the Laboratory Animal Services Centre at The Chinese University of Hong Kong. Animals were maintained on a standard chow (Prolab RMH 2500, 5P14; Lab Diet, St. Louis, MO, United States) and water *ad libitum* up to the time of experiment. Only those diabetic mice with blood glucose levels above 10 mM were included in the diabetic group. Anesthesia before experimentation was achieved with pentobarbitone sodium (50 mg/kg) intraperitoneally. All procedures have been approved by the Animal Experimentation Ethics Committee of the Chinese University of Hong Kong (No. 10/064/MIS).

### 2.2. Isolation of Enterocytes 

Enterocytes were prepared from 4 cm long jejunal segments, beginning 4 cm distal to the ligament of Treitz. Intestinal epithelia cells were isolated and harvested by a Ca^2+^-chelation technique [22]. Briefly, isolated intestinal segments were flushed through with ice-cold saline followed by air. The segment was tied off at one end and filled with Ca^2+^-free hypertonic isolation buffer (7 mM K_2_SO_4_, 44 mM K_2_HPO_4_, 9 mM NaHCO_3_, 10 mM HEPES, 2 mM l-glutamine, 0.5 mM dithiothreitol, 1 mM Na_2_EDTA, and 180 mM glucose, pH 7.4), equilibrated with 95% O_2_ and 5% CO_2_, where over-distention was avoided. The segment was then tied off to form a closed sac and incubated in 0.9% saline at 37 °C with gentle shaking for 16 min. Cells were dislodged manually, and the resulting suspension was collected and centrifuged for 30 s at 500 g; the pellet was re-suspended in freshly prepared cold buffer and this procedure was repeated twice, as we reported previously [23,24].

### 2.3. Western Blot Analysis 

The procedures of Western blot have been described previously [25]. Proteins from the enterocyte and Caco-2 cells were extracted using the CytoBuster protein extraction reagent (Novagen, Darmstadt, Germany) and quantified using Bio-Rad Bradford assay kit (Bio-Rad, Munich, Germany). Proteins (10 μg/lane) were subjected to electrophoresis on a 10% (weight (wt)/volume (vol)) polyacrylamide gel. Proteins from the polyacrylamide gel were transferred to the polyvinylidene difluoride membrane using a semi-dry transblot unit (Bio-Rad, Munich, Germany). The protein-blotted membrane was saturated by submersion in 5% (wt/vol) nonfat skimmed milk in phosphate buffered saline (PBS) (pH 7.4) with 0.1% (vol/vol) Tween-20 for one hour at room temperature. The membranes were incubated with anti-HM74(M-65) receptor rabbit polyclonal antibodies (SC-134583; Santa Cruz Biotechnology, Dallas, TX, United States)(1:100), anti-NIACR1 (Niacin receptor 1) rabbit polyclonal antibodies (NBP1-92180, Novus Biologicals, Littleton, CO, United States) (1:100), and anti-β-actin mouse polyclonal antibodies (EMD Millipore, Danvers, Massachusetts, United States) (1:5000) overnight at 4 °C. After washing with 0.1% Tween-20 in Phosphate-buffered saline (PBS), membranes were incubated with the following corresponding peroxidase-labeled secondary antibodies for one hour at room temperature: anti-rabbit Immunoglobulin G (IgG) antibody (GE Healthcare Bio-Sciences, Pittsburgh, PA, United States) (1:1300) and anti-mouse IgG antibody (GE Healthcare Bio-Sciences, Pittsburgh, PA, United States) (1:2500). The positive signal was revealed using Enhanced Chemiluminescence-plus (ECL-plus); GE Healthcare Bio-Sciences, Pittsburgh, PA, United States) western blotting detection reagent and Fuji Medical Super RX-N autoradiography film (FUJIFILM, Valhalla, NY, United States). The intensity of the bands was quantified using FluorChem software (ProteinSimple, San Jose, CA, United States). The corresponding expression of β-actin of each sample was used to normalize its expression of the target protein.

### 2.4. Real-Time PCR Analysis 

Quantitative RT-PCR was performed using StepOne™ Real-Time PCR Systems (ThermoFisher Scientific, Grand Island, NY, United States). The procedure has been described previously [26]. Briefly, total RNA was extracted from freshly prepared enterocytes or Caco-2 cells grown in 5.6, 11.2, or 25.2 mM glucose using Trizol reagent (ThermoFisher Scientific, Grand Island, NY, United States) according to the manufacturer’s instructions. RNase Out (ThermoFisher Scientific, Grand Island, NY, United States) was added to the RNA solutions to prevent degradation by RNase. Total RNA served as the template for cDNA preparation using iScript™ Select cDNA Synthesis Kit (Bio-Rad Laboratories, Munich, Germany). Primers were designed from mice and human cDNA sequences using Primer Express Software provided by ThermoFisher Scientific. Mice β-actin and Human β-actin were used as reference genes to normalize the relative expression of each target gene. The sequences of primers used are shown in Table 1. Sybergreen reactions were set up in a volume of 25 μL with ABI two-step sybergreen PCR reagents (ThermoFisher Scientific, Grand Island, NY, United States). Each reaction consisted of 12.5 μL PCR master mix, 0.05 to 0.30 μM of each amplification primer, and 1 μL cDNA. Each sample was run in duplicate with an initial 10-min period at 95 °C to enable the reaction, followed by 40 cycles at 95 °C for 15 s and 60 °C for 1 min. The samples were heated to 60 °C for 1 min, then to 95 °C over the next minute, and finally cooled slowly from 95 °C to 60 °C over 20 min to collect data for the analysis of dissociation curve. Amplification data were collected by the detector of the StepOne™ Real-Time PCR Systems and analyzed with Sequence Detection System software (ThermoFisher Scientific, Grand Island, NY, United States). The threshold cycle (C_T_) of each sample was determined from the time point where fluorescence was first detected, with the cycle number being inversely related to cDNA concentration. The fold changes in mRNA expression were calculated using the 2^−ΔΔCT^ method [27].

**Table 1:** Sequences of specific primers used for real-time quantitative RT-PCR.

Gene	Forward Primer	Reverse Primer
Mouse β-actin (NM_007393.4)	TCCTCCTGAGCGCAAGTACTC	GTGGACAGTAGTGAGGCCAGGT
Mouse GPR109a (NM_030701.3)	GGCGTGGTGCAGTGAGCAGT	GGCCCACGGACAGGCTAGGT
Human β-actin (NC_000007.14)	GGCACCCAGCACAATGAAGATC	ATGCTTCTAGGCGGACTATGACTT
Human GPR109a (NM_177551.3)	TGCCGCCCTTCCTGATGGACA	TGTTCAGGGCGTGGTGGGGA

### 2.5. Immunohistochemistry

Immunofluorescence was carried out as described previously with some modifications to determine the mucosal localization of niacin receptors [23]. Isolated jejunal segments were rinsed with cold saline and then quickly transferred to ice-cold 4% paraformaldehyde (PFA) in 0.1 M PBS (pH 7.4) and incubated at 4 °C overnight. Tissue segments were rinsed with PBS and incubated with 20% sucrose in PBS at 4 °C overnight and later embedded in optimum cutting temperature medium (Tissue-Tek, Sakura Finetek Europe B.V., Zoeterwoude, The Netherlands). Sections (6 μm) were collected on Superforst slides (Gerhard Menzel GmbH, Braunschweig, Germany), and these were boiled in 10 mM citrate buffer for 10 min to retrieve the antigens. Sections were incubated with 1% (wt/vol) bovine serum albumin (BSA) and 6% (wt/vol) normal donkey serum (NDS) (Jackson Immuno Research, West Grove, PA, United States) for one hour at room temperature to block nonspecific antibody binding. The slides were incubated overnight at 4 °C with primary antibody (SC-134583; Santa Cruz Biotechnology, Dallas, TX, United States) (1:100), diluted in PBS with 2% NDS and 0.1% Triton X-100. After three washes with PBS, bound primary antibodies were detected by incubation with their corresponding secondary antibodies labeled with Cyc-3 (Jackson Immuno Research, West Grove, PA, United States) (1:100, diluted with 0.1 M PBS containing 2% NDS) at room temperature for 1 h. Immunoreactivity was captured with a fluorescent microscope equipped with a DC480 digital camera (Leica Microsystems, Buffalo Grove, IL, United States). Human Caco-2 cells were grown on silane-coated cover slips, incubated with ice-cold 4% PFA in 0.1 M PBS (pH 7.4) for 8 min, and washed three times with 0.1 M PBS, followed by the same steps as the immunostaining procedures mentioned above starting from the incubation with 1% BSA and 6% NDS (wt/vol), except that anti-NIACR1 rabbit polyclonal antibodies (NBP1-92180) (1:100) was used as the primary antibody.

### 2.6. Glucose Uptake by Everted Jejunal Sleeves 

Prior to measurements of glucose uptake, long jejunal segments (3 to 4 cm long) were taken 3 cm distal to the ligament of Treitz. Glucose uptake was measured using everted jejunal sleeves [28,29]. In brief, isolated jejunal segments were rinsed with cold saline and everted over a glass rod. The tissue was tied securely to the rod and pre-incubated in gassed carbogen (95% O_2_, 5% CO_2_) bicarbonate buffer (128 mM NaCl, 4.7 mM KCl, 2.5 mM CaCl_2_, 1.2 mM KH_2_PO_4_, 1.2 mM MgSO_4_, and 20 mM NaHCO_3_) without glucose for 4 min at 37 °C followed by 15 min using the same buffer, with 0.1 μM to 2 mM of niacin (Sigma-Aldrich, St Louis, MO, United States). Then, concentrated d-glucose was added to the buffer to make up to a final glucose concentration of 50 mM with 0.2 μCi/mL d-[^14^C]glucose (GE Healthcare Bio-Sciences, Pittsburgh, PA, United States) with trace amounts of 0.1 μCi/mL l-[^3^H]glucose (Sigma-Aldrich, St Louis, MO, United States) to correct for non-specific uptake. After a 2 min incubation, the segments were washed rapidly with ice-cold saline containing 0.3 mM phlorizin with stirring for 1 min. The tissue was removed from the rod, oven-dried, and weighed. The dried residue was incubated with Soluene-350 (Perkin-Elmer, Waltham, MA, United States) at 60 °C for 4 h. Scintillation fluid (Ultima Gold; Perkin-Elmer, Waltham, MA, United States) was added, and counting of radioactivity was carried out. The rate of glucose uptake was calculated as picomole per milligram dry weight intestine per second.

### 2.7. Glucose Uptake by Caco-2 Cells 

Caco-2 cells were purchased from ATCC (catalog no. HTB-37). Cells were grown at 37 °C in minimum essential medium (M2279; Sigma-Aldrich, St Louis, MO, United States) with 20% fetal bovine serum (FBS), 1% nonessential amino acids (#11140; ThermoFisher Scientific, Grand Island, NY, United States), and 1% penicillin-streptomycin and gassed with 95% air–5% CO_2_. Cells were subcultured at ~80% confluence, at a cell density of between 8 × 10^4^ and 1 × 10^5^ cells/cm^2^. The medium was changed every two days, and cells were used 14 days after confluence, when they expressed characteristics of enterocyte differentiation [30,31]. Caco-2 cells grown in media containing 5.6 and 25.2 mM glucose for 14 days after confluence were harvested by Trizol reagent (Thermo Fisher Scientific, NY, United States) for RNA isolation and real-time PCR reaction, and Cytobuster (EMD Millipore, Danvers, MA, United States) for Western blotting. 

Caco-2 cells grown in 5.6 mM glucose were rinsed with 25 °C oxygenated bicarbonate buffer (128 mM NaCl, 4.7 mM KCl, 2.5 mM CaCl_2_, 1.2 mM KH_2_PO_4_, 1.2 mM MgSO_4_, and 20 mM NaHCO_3_) with 10% FBS, 1% penicillin-streptomycin, and 1% nonessential amino acids. Cells were preincubated in gassed (95% O_2_–5% CO_2_) bicarbonate buffer containing 50 mM mannitol for 1 h at 37 °C. The buffer was then replaced with fresh bicarbonate buffer containing 50 mM glucose and 0.2 μCi/mL d-[^14^C]glucose (GE Healthcare Bio-Sciences, Pittsburgh, PA, United States), with or without 0.1 μM to 2 mM niacin. In some experiments, 0.3 mM phlorizin or 0.1 mM phloretin was also present. Cells were washed quickly three times by stirring in ice-cold saline containing 0.3 mM phlorizin. The cells were digested with 0.3 M NaOH, and aliquots were added to scintillation fluid (Ultima Gold; Perkin-Elmer, Waltham, MA, United States). Glucose uptake was measured as disintegrations per min per well of a six-well plate containing a monolayer of Caco-2 cells.

### 2.8. siRNA Knockdown of GPR109a in Caco-2 Cells 

Caco-2 cells grown on six wells plates reached 70% confluence at the time of transfection. One day before the transfection, the medium was changed to one containing no antibiotics. Stock Stealth™ RNA (ThermoFisher Scientific, Grand Island, NY, United States) was diluted to 40 nM with 50 µL Opti-MEM^®^ I Reduced Serum Medium without serum. Lipofectamine^®^2000 (ThermoFisher Scientific, Grand Island, NY, United States) was diluted 50 times with Opti-MEM® I Reduced Serum Medium (ThermoFisher Scientific, Grand Island, NY, United States) and incubated 5 min at room temperature. The diluted Stealth™ RNA and Lipofectamine®2000 was mixed and incubated at room temperature for 10 min. The oligomer-Lipofectamine®2000 complexes were added to each well, followed by incubation of the cells at 37 °C in CO2 incubator for 72 h. The transfected cells were harvested for either protein detection or glucose uptake.

### 2.9. Statistical Analysis 

All results were analyzed using Prism 3.0 software. The data were expressed as means ± standard error of the mean (SEM). Student’s unpaired two-tailed *t*-test and One-Way Analysis of variance (ANOVA) (Tukey’s post-hoc test) were employed to detect significant differences between two groups and among three or more groups, respectively. For all comparisons, *p* < 0.05 was considered statistically significant. For RT-PCR, the C_T_ value of the target gene of a sample was first corrected for the C_T_ value of β-actin, before being statistically analyzed [27].

## 3. Results 

### 3.1. Enterocyte Expression of GPR109a 

**Figure 1:**
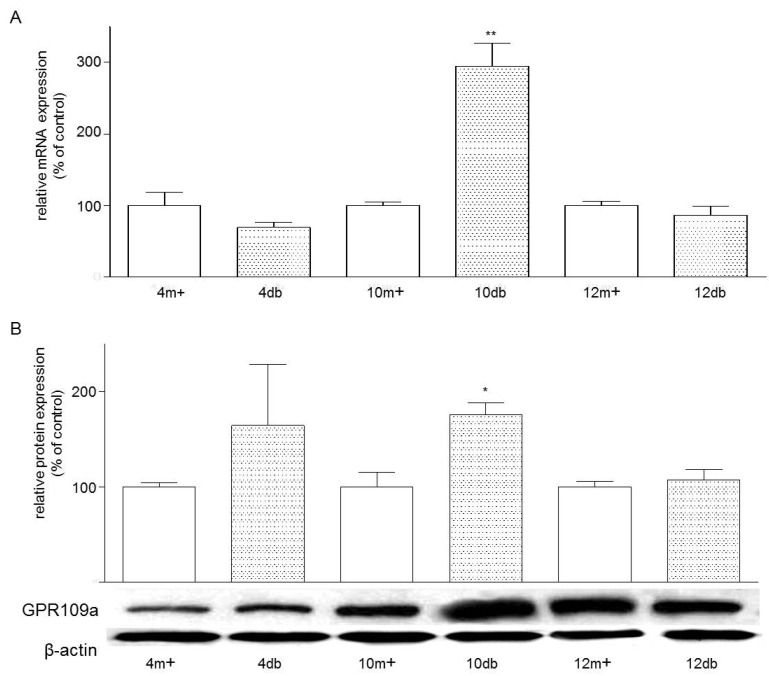
(**A**) Effects of age and Type 2 Diabetes Mellitus (T2DM) on the mRNA expression of GPR109a in jejunal mucosa. Mouse mRNA abundance was determined by real-time PCR using jejunal mucosa and data were calculated as the percentage of their corresponding m+ non-diabetic controls. All results are expressed relative to enterocytes of non-diabetic rats, and are given as mean ± standard error of the mean (SEM), *n* = 3–4; 10 db *vs.* 10 m+, ** *p* < 0.01; (**B**) Effects of age and T2DM on the protein expression of GPR109a in jejunal enterocytes. Western blot analysis showing the relative expression of GPR109a in homogenates of enterocytes prepared from jejuna of *m+/db* and *db/db* mice of 4, 10 or 12 weeks of age. Data were calculated as the percentage change compared to their corresponding *m*+ non-diabetic control. Results are expressed as means ± SEM, *n* = 3–5, 10m+ *vs.* 10 db, * *p* < 0.05. 4m+, 8m+, 10m+ and 12m+ represent 4-, 8-, 10- and 12-week-old *m+/db* mice respectively; whereas 4 db, 8 db, 10 db and 12 db represent 4-, 8-, 10- and 12-week-old *db/db* mice, respectively.

The real-time PCR analysis of mRNA expression of GPR109a normalized to β-actin revealed the presence of GPR109a mRNA in both *m+/db* and *db/db* mouse jejunal brush border (Figure 1A). Expression of GPR109a mRNA in enterocytes from 10-week-old *db/db* mice was higher than that seen in four-week-old *db/db* mice. At the age of 10 weeks, expression of GPR109a in *db/db* mouse jejunum was 2.96 folds greater than that of the control 10-week-old *m+/db* mouse jejunum. Consistently, Western blotting of enterocyte protein also revealed the presence of GPR109a protein in both *m+/db* and *db/db* mice (Figure 1B). Expression levels of GPR109a protein in enterocytes from 10-week-old diabetic *db/db* mice were 1.76 folds greater than their corresponding control 10-week-old *m+/db* mice. 

### 3.2. Localization of GPR109a in Jejunal Mucosa of m+/db and db/db Mice, and Caco-2 Cells 

**Figure 2:**
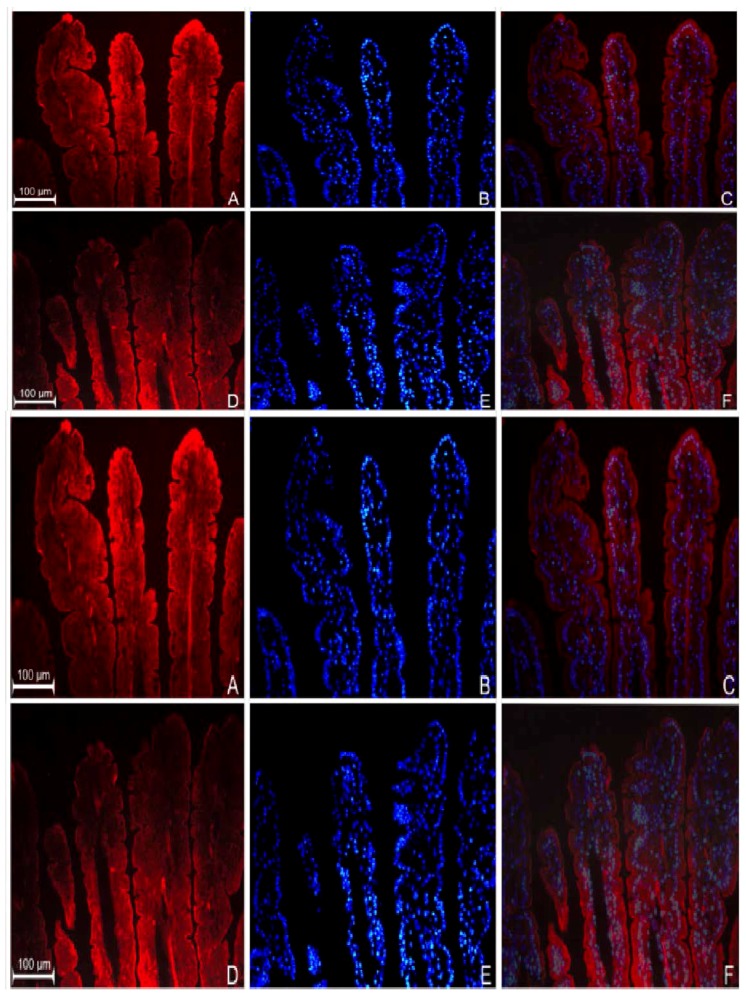
Immunodetection of GPR109a in jejunum of 10-week-old *m+/db* mice (**A**) and 10-week-old *db/db* mice (**D**). (**B**,**E**) are the nuclear DAPI staining of (**A**,**D**) respectively; (**C**,**F**) are their corresponding merged images. Higher magnification of niacin receptor staining of *m+/db* (**G**) and *db/db* (**H**); (**I**) shows jejunal sections prepared in the absence of primary antibodies (negative control). Immunodetection of niacin receptor in Caco-2 cells grown in 5.6 mM (**J**) and 25.2 mM glucose (**M**); (**K**,**N**) show nuclear 4′,6-diamidino-2-phenylindole (DAPI) staining of (**J**) and (**M**) respectively; (**L**,**O**) are the corresponding merged images.

Immunohistochemical results showed the localization of GPR109a at the level of the BBM along the entire jejunal villus length in both normal (*m+/db*) (Figure 2A–C) and diabetic (*db/db*) (Figure 2D–F) animals. However, expression of GPR109a was consistently higher in diabetic jejunum and this was particularly apparent at higher magnification (Figure 2H diabetic *vs.* Figure 2G normal). On the other hand, immunocytochemistry also revealed the presence of niacin receptors at the cell membrane of Caco-2 cells from the 14 days after confluence (Figure 2J–O). In examination, expression of niacin receptor was higher in cells grown in 25.2 mM glucose (Figure 2M) compared to those cultured in 5.6 mM glucose (Figure 2J).

### 3.3. Effects of Niacin on Jejunal Glucose Uptake 

**Figure 3:**
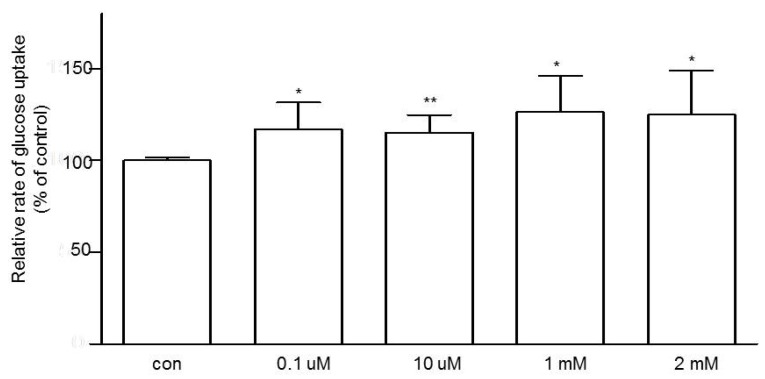
The dose-dependent effect of niacin on jejunal glucose uptake using 10-week-old *m+/db* mice. Data are expressed as relative percentage of glucose uptake relative to the control (con). *n* = 6 for each concentration of niacin. Data are given as means ± standard error of the mean (SEM), * *p* < 0.05 *vs.* control; ** *p* < 0.01 *vs.* control.

Next, we attempted to study the role of GPR109a in modulating jejunal glucose uptake. By using the intestine from non-diabetic (*m+/db*) mice, mucosal exposure to niacin for 10 min increased glucose uptake in a dose-dependent fashion (Figure 3). At 1 mM niacin, glucose uptake was increased by 26.8%. 

### 3.4. Effects of Glucose Concentrations on the Expression of GPR109a in Caco-2 Cells

**Figure 4:**
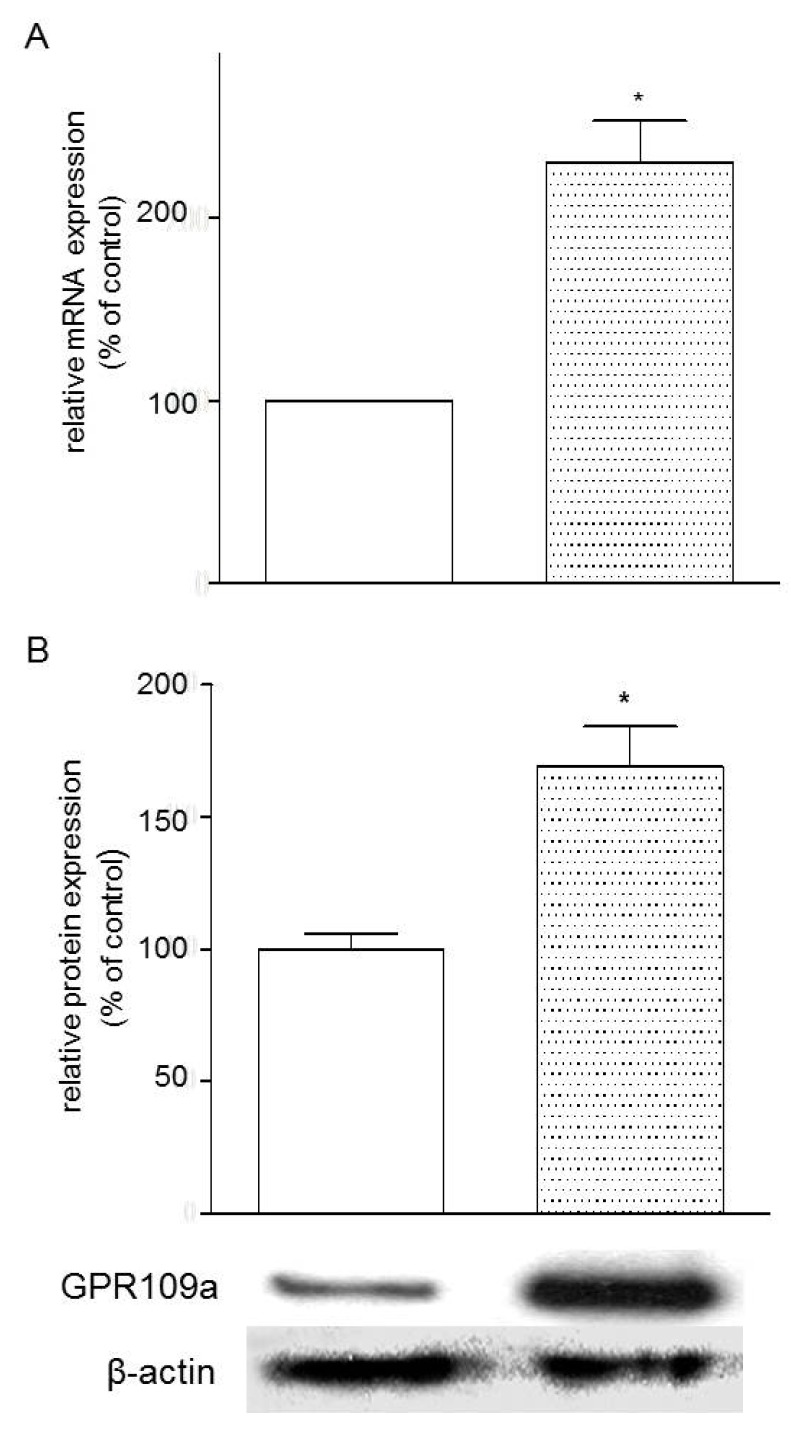
(**A**) Effects of high glucose concentration (25.2 mM) on the mRNA expression of GPR109a in Caco-2 cells *vs.* low glucose concentration (5.6 mM). Results are expressed as means ± standard error of the mean (SEM), *n* = 3, control = 5.6 mM glucose, control *vs.* 25.2 mM glucose, * *p* < 0.05; (**B**) Effects of high glucose (25.2 mM) on GPR109a protein expression in Caco-2 cells *vs.* control 5.6 mM glucose concentrations. Results are expressed as means ± SEM, *n* = 6, control = 5.6 mM *vs.* 25.2 mM, * *p* < 0.05.

Caco-2 cells were used to study the effects of glucose concentrations of the growth medium on the gene expression of GPR109a. GPR109a mRNA was expressed in Caco-2 cells grown in both low glucose and high glucose media. In comparison with the low glucose concentration (5.6 mM), it was observed that high glucose concentration (25.2 mM) increased both of the gene and protein levels of niacin receptor expression by 130.2% and 69%, respectively (Figure 4A, B).

### 3.5. Effects of Niacin and Glucose Transporter Inhibitors on Glucose Uptake in Caco-2 Cells

**Figure 5:**
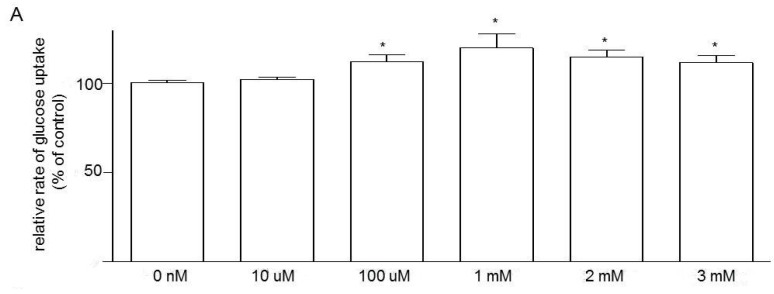

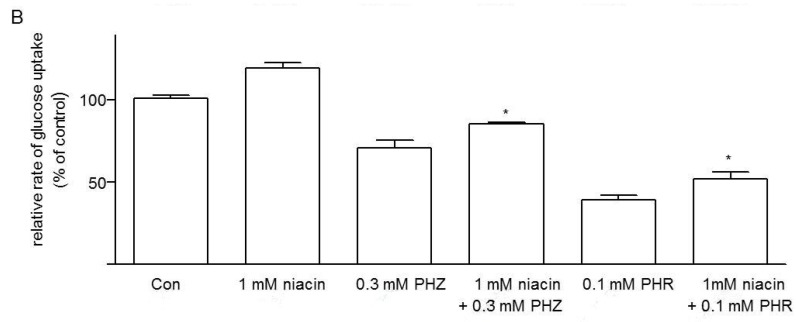
(**A**) Dose dependent effect of niacin on glucose uptake by Caco-2 cells grown in glucose (5.6 mM) (expressed as percentage of glucose uptake relative to their control). *n* = 6 for each concentration of niacin. Data are given as means ± standard error of the mean (SEM), 0 nM *vs.* treatment groups, * *p* < 0.05; (**B**) Glucose uptake by Caco-2 cells grown in 5.6 mM glucose showing effect of 1 mM niacin, with or without phlorizin (PHZ, 0.3 mM) and phloretin (PHR, 0.1 mM). Results are expressed relative to the respective control value (con). All data are given as means ± SEM, *n* = 6. 0.3 mM PHZ *vs.* 0.3 mM PHZ + 1 mM niacin and 0.1 mM PHR *vs.* 0.1 mM PHR + 1 mM niacin, * *p* < 0.05.

We also sought to study the role of the niacin receptor GPR109a in the modulation of glucose uptake in Caco-2 cells. Caco-2 cells grown on normal glucose (5.6 mM) and exposed to niacin for 10 min increased glucose uptake in a dose-dependent fashion (Figure 5A); at 1 mM niacin, glucose uptake was increased by 20.1%. On the other hand, the effects of the two glucose transporter inhibitors, the SGLT1 blocker phlorizin (0.3 mM) and GLUT2 blocker phloretin (0.1 mM), in the presence or absence, on the glucose uptake of Caco-2 cells grown in normal 5.6 mM glucose were also examined. As shown, 0.3 mM phlorizin and 0.1 mM phloretin inhibited glucose uptake by 23.3% and 62.5%, respectively.In addition, 1 mM niacin further increased glucose uptake when present with 0.3 mM phlorizin and 0.1 mM phloretin (Figure 5B). These data indicate that both SGLT1 and GLUT2 may be involved in the upregulation of niacin-induced glucose uptake.

### 3.6. Effects of Knockdown of GPR109a on Glucose Uptake in Caco-2 Cells

**Figure 6:**
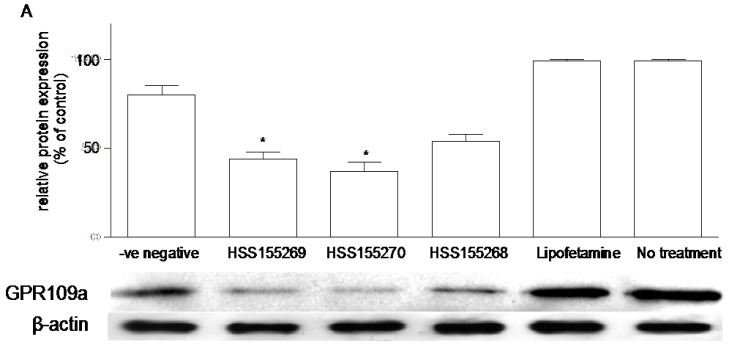

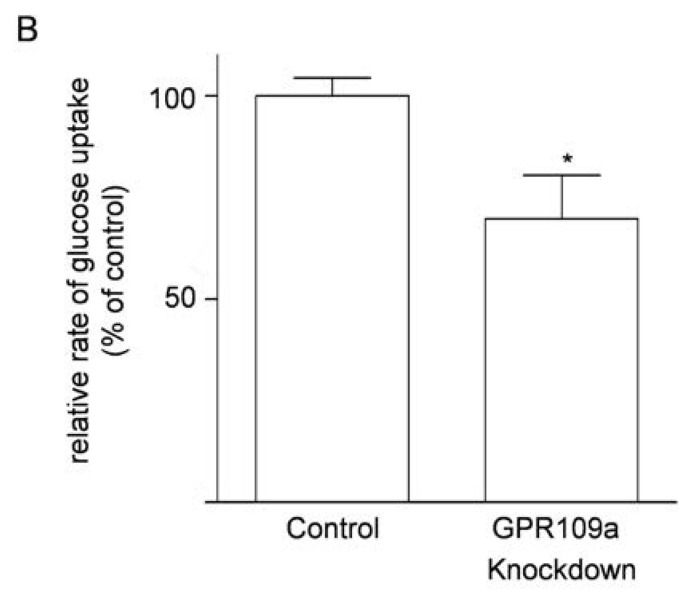
(**A**) Effects of siRNA knockdown of GPR109a on the expression of GPR109a protein in Caco-2 cells grown in 5.6 mM glucose. Western blot analysis showing the relative expression of niacin receptor in homogenates of cells prepared from the -ve negative (negative control siRNA, *Silencer*^®^ Select Negative Control siRNA, non-targeting SiRNA with limited sequence similarity to known genes), and three different sequences of siRNA (HSS155269, HSS155270 and HSS155268). “Lipofetamine” contains only the lipofetamine transfection buffer, without any siRNA. “No treatment” contains neither the transfection buffer nor any siRNA. Results are expressed as means ± standard error of the mean (SEM), *n* = 6. HSS155269 *vs.* negative control, 45% decrease in expression, * *p* < 0.05; HSS155270 *vs.* negative control, 53.8% decrease in expression, * *p* < 0.05; (**B**) Effects of siRNA (HSS155270) knockdown of GPR109a on the glucose uptake of Caco-2 cells grown in 5.6 mM glucose. Knockdown of GPR109a decreased the rate of glucose uptake by 30.2%, *n* = 5, * *p* < 0.05.

Three different siRNA sequences (HSS155268, HSS155269 and HSS155270) were tried in the study of knockdown of niacin receptor in Caco-2 cells grown in 5.6 mM glucose. Western blot analysis showed that the knockdown experiments with siRNA HSS155269, HSS155270 and HSS155268 decreased protein expression of niacin receptors by 45.0%, 53.7%, and 32.5%, respectively as compared with the negative siRNA control group (Figure 6A). Therefore, we selected the siRNA HSS155270, the most effective knockdown sequence, which decreased the rate of glucose uptake of Caco-2 cells grown in 5.6 mM glucose by 30.2% (Figure 6B). 

## 4. Discussion 

Niacin is a commonly employed anti-hyperlipidemia drug but it is also widely known to induce hyperglycemia during chronic and high-dose therapy [5,6]. Despite this, the mechanistic action of niacin and its receptor GPR109a in the regulation of glucose homeostasis, in particular with intestinal glucose uptake, remains ambiguous. The present study has shown, for the first time, that enterocyte GPR109a is expressed and functional in the local control of intestinal glucose absorption, and that type 2 diabetic and high glucose conditions upregulate the GPR109a expression, thus enhancing niacin-induced intestinal glucose uptake in the mice. Our laboratory has recently reported the novel role of niacin, via its activation of GPR109A, in pancreatic islets [10]. In that study, niacin was found to increase fasting glucose concentrations, reduce glucose-stimulated insulin secretion (GSIS) and insulin secretion, and impair glucose tolerance, thereby inducing pancreatic islet dysfunctions in high-fat diet (HFD) induced obese mice [10]. In this regard, the present study establishes a previously undiscovered role of niacin in local regulation of glucose homeostasis at the level of the small intestine in normal and diabetic states.

In the present study, we have first demonstrated that the mRNA and protein expression levels of the niacin receptor GPR109a were elevated in the diabetic *db/db* mice, as evidenced by real-time qPCR and Western blot, as well as further supported by the immunohistochemical results. Interestingly, an upregulated expression GPR109a was also observed in diabetic mouse and human retina, where GPR109a could serve as an anti-inflammatory receptor in retinal pigement epithelial cells and is closely associated with diabetic retinopathy [32]. It is well recognized that inflammatory process is a key causative factor in the pathogenesis of diabetes and GPR109a has been shown to be a potent anti-inflammatory receptor in various physiologically important organs, including but not limiting to, the colon [33], blood vessels [34], and pancreas [10]. In light of these findings, it prompts us to speculate that such an elevated GPR109a expression may be responsible for acting as a strategic counteractive mechanism against the inflammatory insults, as induced by hyperglycemia in diabetic states.

To address this issue, we sought to examine whether niacin could locally regulate glucose uptake across the small intestinal BBM. In fact, our laboratory has previously shown the functional existence of a local renin-angiotensin system (RAS) in the regulation of intestinal glucose uptake, where binding of the two physiologically active components of this system, angiotensin II, to its angiotensin type 1 receptor and the binding of angiotensin (1–7) to the Mas receptor at the jejunal BBM in enterocytes inhibit SGLT1-mediated glucose transport across this membrane in a dose-dependent fashion [23,35]. Therefore, we explore to investigate whether niacin is also able to regulate glucose uptake locally in the intestine. First of all, it is necessary to address if niacin arriving at the small intestine would be adequate to elicit its physiological function. Studies have shown that niacin is indeed equally absorbed by the stomach and the upper small intestine in human [36]. In fact, up to about four grams of niacin could be almost completely absorbed by adults. Intestinal luminal concentration of niacin under physiological conditions has been reported to lie in the micromolar but not in the millimolar range [37]. As a result, there should be sufficient amount of niacin reaching the jejunum for its physiological actions. Our results showed that niacin increased intestinal glucose uptake in *m+/db* mice significantly and dose-dependently; these data were further substantiated by the observed decrease in glucose uptake after GPR109a knockdown in Caco-2 cells. Our findings are in line with the observation that niacin treatment increases glucose uptake in adipocytes where such an effect of niacin on glucose uptake was antagonized by theophylline and isoprenaline, which are that agents that increase intracellular concentrations of 3′,5′-cyclic adenosine monophosphate (cAMP); in addition, niacin could further increase the maximum rate of glucose transport stimulated by insulin in adipocytes [38]. In adipocytes, niacin treatment significantly lowered the intracellular concentrations of cAMP in adipocytes, and a decrease in cAMP level upregulated glucose uptake [38,39]. However, cAMP has been shown to stimulate SGLT1 and thus mediated intestinal glucose uptake in enterocytes [40], thereby contradicting the findings in adipocytes. Not withstanding these findings, it might be plausible to postulate that cAMP is a downstream signaling molecule of GPR109a for its subsequent mediation of glucose uptake. Despite this, further investigations are warranted in order to confirm the precise role of cAMP in jejunal enterocytes.

Apart from the *in vivo* data, we further investigated the effects of high glucose treatment on the expression profiles of mRNA and protein of GPR109a in human Caco-2 cells. Our data revealed that 25.2 mM glucose concentration raised remarkably the mRNA and protein levels of GPR109a, when compared to those of the Caco-2 cells grown in 5.6 mM glucose. To address which glucose transporters are involved in GPR109a-mediated intestinal glucose uptake, we sought to add phlorizin (SGLT1 blocker) and phloretin (GLUT2 blocker) to examine whether the stimulatory effect of niacin on glucose uptake was mediated via SGLT1 and/or GLUT2. In this regard, we found both SGLT1 and GLUT2 were responsible for this regulatory process. In terms of SGLT1, cAMP has been shown to stimulate SGLT1 function in enterocytes as mentioned above [40] and involved in post-transcriptional stabilization of SGLT1 message [41]. Therefore, it is plausible to propose that cAMP may act as a downstream mediator for the action of niacin on SGLT1-mediated glucose uptake. In term of GLUT2, however, cAMP has been previously reported to prevent glucose-mediated stimulation of GLUT2 in hepatocytes [42], and thus a decrease in cAMP level in hepatocytes is supposed to upregulate GLUT2-mediated glucose uptake; however, this postulate is apparently contradictory with that of SGLT1. In view of this, the signaling molecules rather than GLUT2 might be involved in the regulation of niacin-stimulated GLUT2 activity, which merits intensive investigations in the future.

Besides, we also questioned whether there a crosstalk among GPR109A, SGLT1, and GLUT2 might exist. In this context, niacin binding to GPR109A has been shown to increase the Protein Kinase C (PKC) activity in CHO-K1 cells [43]. Furthermore, GLUT2 can be PKC-dependently translocated apically upon high glucose exposure [44]. In the pancreatic islet, activation of Protein Kinase A (PKA) by adenylyl cyclase inhibited the GLUT2 mediated initial rate of 3-*O*-methyl glucose uptake by 48% [45]. Also, activation of PKC has been shown to activate human SGLT1 [46]. In the apical GLUT2 model, SGLT1-mediated glucose transport promotes insertion of GLUT2 into the apical membrane within minutes, where the capacity of GLUT2 mediated glucose transport can be increased up to more than three fold [21]. On the grounds of the evidence, it might be possible that niacin binding to GPR109a inhibits the PKA activity, and activates PKC activity at the same time. The niacin binding might increase the translocation of GLUT2 to the apical membrane, thus GLUT2-mediated glucose uptake. Another possibility is that GPR109a activation by niacin effects the production of SGLT1. In the uptake experiment, the time of the niacin treatment lasted only few minutes. The time may not be long enough to induce the upregulation of SGLT1 expression. Since an increased SGLT1 activity would upregulate GLUT2 activity [21], the increase in GLUT2 activity by niacin binding to GPR109a might upregulate SGLT1 mediated glucose uptake.

## 5. Conclusions

In conclusion, the present study is the first to report that niacin is able to bind to the niacin receptor GPR109a to stimulate SGLT1- and GLUT2-mediated jejunal glucose uptake in hyperglycemia observed in diabetes. Given niacin is a crucial nutritional supplement that garners attention over the recent years, our results should provide a clinical implication that the niacin receptor GPR109a may serve as a potential therapeutic target for novel dietary or pharmacological approaches to controlling intestinal sugar delivery, thereby improving glycemic control for diabetes.
